# Facial skin characteristics and concerns in Indonesia: A cross‐sectional observational study

**DOI:** 10.1111/srt.13189

**Published:** 2022-07-04

**Authors:** Yaping Du, Chandraprabha Doraiswamy, Jie Mao, Qian Zhang, Yan Liang, Zheng Du, Renuka Vasantharaghavan, Manoj Kumar Joshi

**Affiliations:** ^1^ Unilever R&D Shanghai Shanghai China; ^2^ Unilever R&D Bangalore Bangalore India

**Keywords:** aging, ethnicity, facial skin attributes, skin conditions

## Abstract

**Background:**

Facial skin characteristics and appearance vary according to ethnicity. While much of this knowledge is derived from the Caucasian population, lately there have been efforts to gain such understanding in various regions in Asia.  In this paper, we have built an understanding of such features in Indonesia. In Indonesia, a section of females wears a traditional veil (hijab) to cover the scalp and part of face. The influence of the hijab on facial skin attributes was also investigated.

**Methods:**

In a cross‐sectional observational study design involving 419 female volunteers in Jakarta, Indonesia, facial skin attributes (colour, radiance, hydration, trans‐epidermal water loss [TEWL], wrinkles, fine lines, pores, and sebum levels) and conditions (melasma, post‐inflammatory hyperpigmentation (PIH), solar lentigines/ senile lentigines, seborrheic keratoses and acne) were assessed by trained operators and dermatologists using standard validated instruments and scales.

**Results:**

With age, facial skin colour showed darkening in cheek; forehead on the other hand showed slight lightening. The skin evenness and radiance decreased, substantially. Aging attributes measured in terms of lines, wrinkles, and under‐eye dark circles showed deterioration with age; the decline was progressively faster than colour change. Facial image data analysis corroborated these findings. Skin hydration remained similar across the age groups even though the skin barrier function measured in terms of TEWL improved with age. Sebum levels in the skin were similar up to the age of 50 but declined in the next group of 50–60 year. Pore severity increased with age. Melasma, seborrheic keratosis and PIH showed a high prevalence (>∼50%) at the young age group (20–30 years), itself. Melasma prevalence attained 100% in the age group of 41–50 year and onwards, and its severity similarly showed a steady rise with age. PIH on the other hand showed a steady decline with age. Solar lentigines prevalence (∼30%) did not change much across age groups, and the severity scores were similar in age groups up to 50 year but increased substantially in 51–60‐year age groups. Seborrheic keratosis was similar (∼47%) in age groups up 20–40 year but steadily increased in upper age groups. Its severity was similar in the age groups of 20–30 year and 31–40 year but showed a two‐fold increase in subsequent age groups. Acne was 10% in the age group of 20–30 year and declined gradually to 0.7% in the 51–60‐year age group. Hijab wearers showed slight protection in skin colour darkening and improvement of evenness and radiance but were similar on aging (fine lines and wrinkles on crow's feet, under eye and peri‐oral areas) markers to non‐wearers. In general, in majority of age‐groups, hijab wearers showed a higher prevalence of melasma, solar/senile lentigines, seborrheic keratosis and PIH.

## 1 INTRODUCTION

Skin characteristics, and the impact of age on them, vary according to geographical location, genetics, lifestyle, and ethnicity.^1^, ^2^, ^3^, ^4^, ^5^, ^6^, ^7^, ^8^, ^9^, ^10^ In Asia, there have been studies from Korea,^11^, ^12^ Japan,^13^, ^14^ China,^15^ multiple Asian cities (China, India, South Korea, Japan, and the Philippines)^7^ and India.^9^ In this study, we have generated such an understanding for Indonesian females living in Jakarta, Indonesia. A section of females in Indonesia partially covers their scalp and face using a traditional veil (hijab). For gaining a comprehensive understanding of skin concerns and to learn the influence of hijab on skin characteristics in Indonesian females, we conducted a dermatologist‐assisted systematic characterization of facial skin in Jakarta. The study involved visual evaluations by trained dermatologists and instrumental evaluations of colour, pigmentation, and aging. These measurements have widely been shown to reflect on the state of skin health and conditions.^7^

## 2 MATERIALS AND METHODS

### 2.1 Study subjects and ethical review

This study involved a cross‐sectional observational design with no product or behavioural intervention and conducted between 26 June 2018 and 31 August 2018 in Jakarta, Indonesia. Of 420 females recruited, 419 people finally completed the investigation. One subject lost to follow‐up due to personal reasons. The subjects, in the range of 20–60 years (Y) (both included), were staggered in a 10‐Y cluster with 100 subjects each starting from year 20–30 to 51–60 Y (age groups 20–30 Y, 31–40 Y, 41–50 Y and 51–60 Y) with approximately 50 subjects per 5‐year intervals. In each 10‐Y cluster, the subject number of hijab wearers and non‐hijab wearers was also balanced. Informed consents were obtained from subjects who met the qualifying criteria and were willing to participate in the study. Study protocol was approved by the ethics committee of the Faculty of Medicine, University of Indonesia.

Four hundred twenty Indonesian female subjects were screened to ensure 400 female subjects completed the study. The selection of suitable subjects was made according to the inclusion and exclusion criteria described in the following sections.

#### 2.1.1 Inclusion criteria

1. Be Indonesian female, between the ages of 20 and 60 inclusive and be in general good health.
2. Understand the test procedure, read and sign an appropriate informed consent form indicating their willingness to participate.
3. Be willing to be photographed for facial images and agreeing that images can be used further research analysis/evaluation by the study team.

#### 2.1.2 Exclusion criteria

1. Subject who was pregnant, trying to be pregnant or breast feeding or has had a baby within the last 12 months.
2. Subject having done facial injections and/or aesthetic surgery.
3. Be involved in any aspect of test administration, that is, evaluating or overseeing activities related to product.
4. Have participated in any clinical study involving the test sites in the past 3 months or is subject participating in any clinical study concurrently.
5. Have a history of any type of cancer, skin disease, any skin condition on the test sites, or currently taking a medication that the investigator feels would interfere with the study.
6. The subject is an employee of Unilever or the site conducting the study.

### 2.2 Study design and clinical measurements

Assessments at each visit: Subjects were assessed in three independent visits at the assessment site. In the first visit, they were assessed against study screening criteria, provided their health history, and signed informed consent if found eligible for the study.

In the second visit, subjects reported without wearing make‐up or application of any skin care product in last 24 h. They also refrained from the use of any skin care product during the visit. Subjects cleaned their face with mild soap‐bar and waited for at least 15 min in the temperature and humidity‐controlled room (20–22°C, 45%–55% humidity) before any further assessment.

Images of each subject's face (the front, right and left sides from 45‐degree angle) were captured by VISIA‐CR (Canfield Scientific, Inc. USA). The images were captured under five lighting modes, including S1, S2, CP, PP and UVNF.

Subsequently, the subjects were evaluated by trained dermatologists for the skin colour, radiance, hyper‐pigmentation, evenness, pores, lines, and wrinkles, melasma, solar lentigines, seborrheic keratoses, post‐inflammatory hyperpigmentation (PIH) and under eye dark circles (UEDCs). Skin colour types were determined in terms of individual typology angle (ITA)‐based classification according to Chardon et al.^16^ and Del Bino and Bernerd.^17^ For visual evaluation of various skin attributes, Unilever proprietary scales were used unless mentioned otherwise. Pores and melasma severity were measured as per Huixia et al. ^18^ and Pandya et al.,^19^ respectively. Next, five non‐invasive instrumental measurements were taken in the given order ensuring a minimum 1 h of equilibrating at 20–22°C, 45%–55% humidity in a temperature and humidity‐controlled room, (1) skin firmness on both sides (right and left) of the side cheek using a cutometer (MPA 580, Courage + Khazaka, Koln, Germany); (2) skin colour (L*, a*, b*) on both sides of forehead and upper cheek by skin reflectance Spectrophotometer CM2600d (Minolta, Osaka Japan); (3) Trans‐epidermal water loss (TEWL) on each cheek using Derma lab (Cortex Technology, Denmark); (4) skin hydration on each of the upper cheek using Corneometer CM 825 (Courage + Khazaka Electronic GmbH) and (5) skin sebum on sides of the forehead using sebumeter (Courage + Khazaka Electronic GmbH). All the above measurements were taken within 1.5 h after face wash at the clinical site. The measurements for sebum were taken 6 h after washing the face. All the measurements involved trained operators who conducted them using standard operating procedures and calibrated and validated instruments.

In 3rd and final visit lasting around 30 min, subjects underwent a visual dermatologist's assessment for skin disorders (hyperpigmentation of genetic origin and acne [*Acne vulgaris*]). A chronic inflammatory disorder of the pilosebaceous origin, acne presents itself in multiple forms such as comedones, papules, pustules, and nodules. They may have diverse triggers and could attain various developmental stages expressed as grade I to IV. In this study, dermatological assessment included all these forms.

### 2.3 Quantitative image analysis

Images of whole face were captured using the VISIA CR photography system. Unilever proprietary image analysis was applied to detect and quantify the line/wrinkle and hyperpigmentation features from the VISIA CR images after removing non‐skin components and background. For wrinkle measurement, a detection method based on the Fourier‐transform allowed calculation of wrinkle coverage as percent pixels per area, whereas a Gaussian filtering method resulted in the lines and wrinkle map contrast weighted area (CWA). Mottled hyper‐pigmentation % was calculated as percent of locally dark areas of image with respect to total image area to represent overall evenness. Blemish map CWA was determined to indicate the blemish visibility (both contrast and intensity) of pigmented spots.

### 2.4 Statistical analysis

Descriptive statistics (*n*, mean, SD, SE, 95% confidence intervals) for each endpoint was calculated and presented by age and hijab groups. Mixed model with raw values as response, age group and location as fixed effects, an interaction effect of age group and facial location to observe the combined change of endpoints over age groups at each location considered and study subjects as random effect was performed. Further a regression model for each location was fitted, considering age groups as a factor to understand the intrinsic contribution of the endpoints at each location. All statistical analyses were performed at 95% confidence interval using SAS 9.4.

## 3 RESULTS

Attributes related to facial skin colour, aging pattern and skin conditions were measured by trained dermatologists or operators using validated instruments or scales. The study also included subjects who practiced wearing hijab. The impact of this practice influencing the skin attributes at different age groups was assessed.

### 3.1 Skin colour

Facial skin colour was measured at forehead and cheeks across age groups visually by trained dermatologists using a skin colour scale (range 1–14 with 1 shade being the lightest). Instrumental measurements of colour were made using the commission Internationale de I'eclairage (CIE) 1976 L*a*b* colour system, where L* represents skin lightness, and a* and b* are chromaticity coordinates representing the colour saturation from green to red and blue to yellow, respectively.^20^ A change in L*, a* and b* from their baseline values has been shown to indicate lightness, red and yellow (sallowness) tone of the skin (Galzote et al., 2013^7^).

**FIGURE 1:**
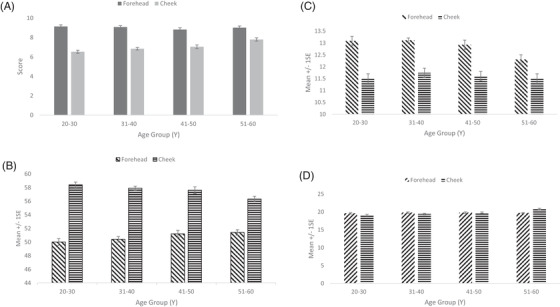
Facial Skin colour across age groups. (A) Visual colour assessment. (B) Spectrophotometric L*. (C) Spectrophotometric a*. (D) Spectrophotometric b*

Visual scores for skin colour in forehead remained similar across age groups (Figure 1A), although the L* showed a slight increase in age groups of the 41–50 Y and 51‐60 Y (Figure 1B). a* showed (Figure 1C) a decrease of 0.8 units in 51–60 Y age group over the lower age‐groups. b*, on the other hand, remained unchanged across age groups (Figure 1D). The visual scores on cheeks showed a steady increase with age; there was a 19% increase in scores in age group 51–60 Y compared to the 20–30 Y. L* showed a trend like visual scores with a decrease of 2.1 units in age group of 51–60. a* remains unchanged. b* showed an increase of 1.9 units in the 51–60 Y in comparison to the 20–30 Y age group.

ITA‐based skin colour type distribution of the study population is shown in Table 1. It ranged from skin colour type III (intermediate) to type V (brown) with predominantly being type IV (tan).

**TABLE 1:** Skin biophysical parameters and sebum levels across age groups

		Age group (Y)				
Skin biophysical parameters (mean ± SE)		20–30	31‐40	41‐50	51‐60	*p*‐Value
Skin hydration		49.7 ± 1.6	47.5± 1.6	49.1 ± 1.3	47.6 ± 1.5	0.4768
TEWL (gm/m^2^/h)		9.89 ± 0.66	9.1 ± 0.57	9.05 ± 0.71	6.45 ± 0.41	0.0002
Sebum (μg/cm^2^)		154.7 ± 8.7	160.2 ± 7.8	152.9 ± 11.6	104.6 ± 11.5	0.0008
Skin elasticity	R2	0.63 ± 0.01	0.57 ± 0.01	0.52 ± 0.01	0.46 ± 0.01	<0.0001
	R7	0.35 ± 0.01	0.30 ± 0.01	0.26 ± 0.01	0.22 ± 0.005	<0.0001

Abbreviation: SE, Standard Error.

### 3.2 Facial skin radiance and evenness

**FIGURE 2:**
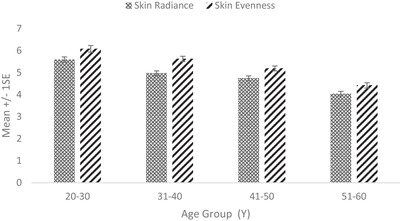
Facial skin radiance and evenness across age groups

Facial skin radiance measured by trained dermatologists (using a scale of 1–9 with 1 being the lowest) showed a steady decline with age. The radiance value was 27% lesser in the 51–60 Y age group than 20–30 Y (Figure 2). Skin colour evenness (measured using a scale of 1–9 with 1 being lowest) showed a similar pattern (Figure 2).

### 3.3 Aging attributes

**FIGURE 3:**
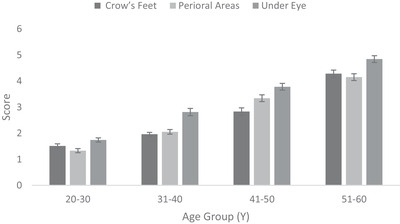
Facial fine lines and wrinkles (visual) on crow's feet, under‐eye, and peri‐oral area across age groups

Fine lines and wrinkles on crow's feet, under‐eye and peri‐oral area measured using a 0–9 scale increased steadily with age such that the 51–60 Y age group showed over two‐fold increase in comparison to youngest age group (Figure 3).

### 3.4 Skin biophysical parameters

**FIGURE 4:**
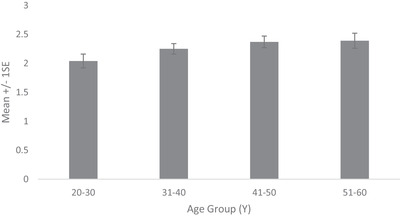
Facial pore severity across age groups

Skin attributes measured using biophysical techniques are shown in Table 2. Skin hydration, measured in terms of skin capacitance values, was similar across age groups.  The TEWL on the other hand showed a decrease with age. The decline was 10% in the age groups of 31–40 Y and 41–50 Y and 35% in 51–60 Y age group. Sebum levels were similar in the age groups up to 50 Y but decreased by 30% in higher age group. Two of the applied measures of skin elasticity (R2 [gross elasticity] and R7 [recovery after deformation]) showed a steady decrease with age. R2 showed a decline of 27% in age groups of 51–60 Y in comparison to 20–30 Y whereas such decline was 37% for R7. Pore severity measured in terms of pore size increased with age reaching to its maximum at the 41–50 Y age group. Age groups of 41–50 Y and 51–60 Y showed 15% higher pore severity than age group 20–30 Y (Figure 4).

**TABLE 2:** Skin colour type distribution of study population as per individual typology angle (ITA) (Del Bino and Bernerd^17^)

Skin type	Colour classification	*n* (%)
III	Intermediate	21 (5.0)
IV	Tan	296 (70.7)
V	Brown	102 (24.3)

### 3.5 Image parameters

**FIGURE 5:**
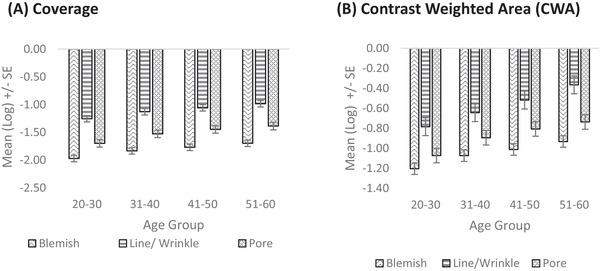
Facial VISIA CR imaging, (A) coverage and (B) contrast weighted area (CWA)

VISIA images were processed (Figure 5) and the coverage parameters such as the Fourier transformed map (FTM) were determined. Total coverage measured the percentage of pixels with respect to the lines and wrinkles. The blemish and pore coverage measured the intensity of these parameters on the skin. These parameters were analysed on the log transformed values. Under coverage parameters, the blemish coverage, FTM total coverage and pore coverage were assessed, and an increase of approximately 15% in the 50–60 years was observed in all the coverage parameters as compared to the 20–30 Y. The CWA parameters followed the similar trend as the coverage parameters.

### 3.6 Dermatologist assessment of skin conditions

**FIGURE 6:**
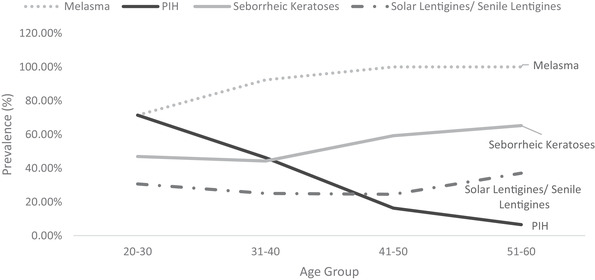
Facial dermatological disorders prevalence (%) across age groups

**FIGURE 7:**
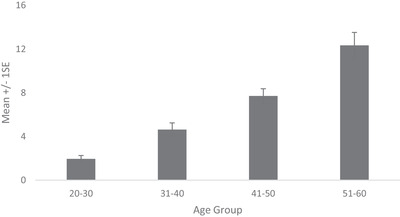
Melasma (MASI scores) across age groups

**FIGURE 8:**
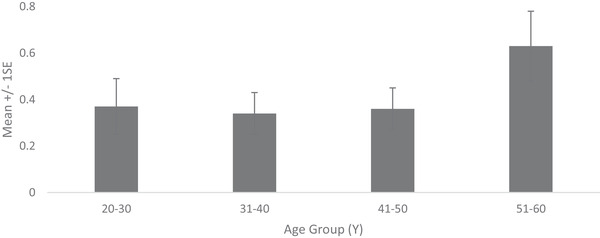
Solar lentigines /Senile lentigines severity across age groups

**FIGURE 9:**
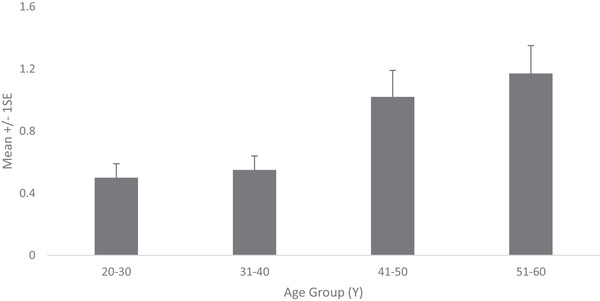
Seborrheic keratoses severity across age groups

**FIGURE 10:**
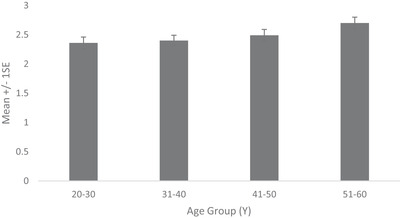
Under‐eye dark circle severity across age groups

Prevalence of dermatologist identified disorders related to skin conditions—melasma, PIH, seborrheic keratoses and solar and senile lentigines—is shown in Figure 6. Melasma prevalence was found to be high from early age. Being 71% in the age group 20–30 Y, it increased to 92% in age group 31–40 and reached 100% thereafter. Melasma severity similarly showed a steady rise with age (Figure 7). Being at melasma area and severity index^18^ of 1.95 in the age group of 20–30 Y, it rose to 4.63, 7.71 and 12.34, respectively, in age groups of 31–40, 41–50 and 51–60. Solar lentigines/senile lentigines prevalence declined from 31% in the age group 20–30 Y to 25% in 31–50 Y but increased again to 37% in the 51–60 Y group (Figure 6). The severity scores were similar (0.4) in the age groups of 20–30 Y, 31–40 Y and 41–50 Y groups but increased to 0.6 in the 51–60 Y age group (Figure 8). Seborrheic keratoses prevalence was similar (47% and 44%, respectively) in the age groups of 20–30 Y and 31–40 Y (Figure 6). It increased to 59% and 65% in the higher age groups. In terms of severity, seborrheic keratoses doubled from being 0.5 at the age up to 40 Y to 1 in later age groups (Figure 9). The PIH prevalence decreased with age, being lesser in older age groups (7%, 16%, 46% and 71% in the age groups of 51–60 Y, 41–50 Y, 31–40 Y and 20–30 Y, respectively) (Figure 6). Acne prevalence was 50%, 23%, 8.0 % and 2 % in the age groups of 20–30 Y, 31–40 Y, 41–50 Y and 51–60 Y, respectively. Under‐eye dark circles measured using a scale of 0–3 showed an increase with age (Figure 10) such that the age group 51–60 Y showed a 14% increase in severity as compared to the younger age group (20–30 Y).

### 3.7 Age dependence of facial skin attributes

In forehead, L* skin colour and a* skin colour showed age dependence. In cheek area, in addition visual, and b* skin colour also showed such dependence, while a* did not. Also, the skin evenness, skin radiance, the aging parameters, wrinkles and lines measured at the Crow's feet, perioral areas and under eye showed a statistically significant effect of age groups. Similarly, the biophysical parameters R2, R7, TEWL, sebum level and pore severity had a significant age‐group effect. The skin disorders such as melasma and seborrheic keratoses also showed the evidence of effect of age groups.

### 3.8 Influence of hijab practice on facial skin characteristics

Facial skin attributes (skin colour, evenness, radiance, lines, wrinkles, UEDC, hydration, TEWL and sebum levels) of hijab wearers were also measured (data not shown) and compared to those not wearing hijab. Visual skin colour of forehead in hijab wearers was slightly lower in all age groups other than the age group 51–60 Y where it was similar. L*measurements corroborated these trends. a* was lower in hijab wearers in the age group of 20–30 Y and 31–40 but similar in other age groups. b* was similar in hijab wearers and non‐wearers across age groups. In cheeks, visual colour showed slight lowering across age groups. However, such lowering was only observed in terms of L* increase in the age group of 20–30 Y and 41–50; in other age groups L* showed no change. Similar to what was observed for forehead, a* in cheek was lower in hijab wearers in age group of 20–30 Y and 31–40 but similar in other age groups. Also, similar to forehead, b* was similar in various age groups in both the groups. The mean scores of radiance and evenness showed a 0.3 units increase over non‐hijab wearers, the difference was high at the younger age groups and tapered off at 50–60. Lines and wrinkles did not show any change across age groups based on hijab status. The VISIA image parameters shared a similar trend (data not shown). UEDC severity showed a steady increase with age in group without hijab (Figure 10). Such increase was arrested in hijab wearing group. Mean value of TEWL was lower in non‐hijab group than hijab wearers across age groups except in the age group “41–50,” indicating better skin barrier function in non‐hijab group. Hydration, sebum levels and pore severity across age‐groups were not affected by hijab. ​

**FIGURE 11:**
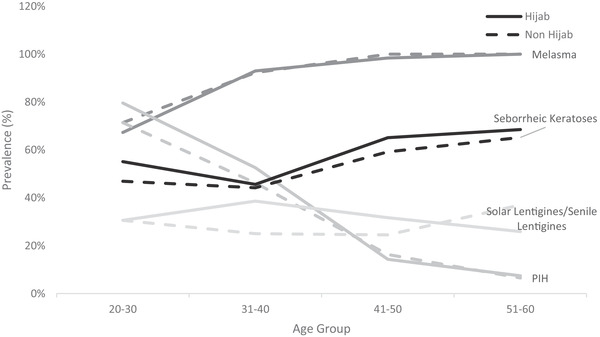
Prevalence of dermatological disorders in hijab and non‐hijab wearers

Influence of wearing hijab on skin conditions is shown in Figure 11. The combined population of all age groups in hijab wearers and non‐wearers showed 32% and 29% prevalence of solar lentigines/senile lentigines, respectively. In the individual age groups, the pattern however varied. The prevalence was similar in the age group of 20–30 Y. The hijab wearers, however, showed 13.6% and 7.2% increase compared to that observed in the non‐hijab group in 31–40 Y and 41–50 Y age group, respectively. In the 51–60 Y age group, on the other hand, hijab wearers showed 11.1% lesser incidence than their counterparts.

For seborrheic keratoses, the combined age groups of hijab and non‐hijab showed prevalence of 59% and 54%, respectively. Hijab wearers showed 8.1%, 3.9% and 3.3% more prevalence for seborrheic keratoses in the age groups of 20–30 Y, 41–50 Y and 51–60 Y, respectively. The prevalence was similar in the 31–40 Y age group.

All age groups combined, hijab wearers and non‐wearers showed similar (37% and 36%, respectively) PIH prevalence, respectively. Analysed age group wise, the prevalence was 8.2% and 6.4% and higher in age groups of 20–30 and 31–40, respectively. In the subsequent age groups, the prevalence was similar.

All age groups combined, melasma prevalence at 91% was similar in hijab and non‐hijab groups. Also, the age‐group wise prevalence was not influenced in hijab group. In hijab group, L* and a* skin colour, UEDC and pore severity lost the effect of age groups that was observed in the group not wearing hijab.

## 4 DISCUSSION

Using multiple well‐established measures of skin characteristics, we measured the facial skin attributes in female subjects of various ages in Indonesia. These attributes were also studied in subjects who wore hijab and that revealed the impact of this habit on the facial skin.

In line with that reported for other ethnicities,^6^ skin colour on the forehead in this study was also darker and redder than cheeks. A comparison of L* values of Jakarta females with other Asian cities showed lower L* (darker skin tone) than Harbin, Guangzhou, Shanghai in China, Seoul, and Sendai, in Korea. The values were like Manila, Philippines, but higher (lighter skin tone) than Calicut and New Delhi.^7^ Skin colour on the forehead with age showed a slight lightening as picked up by spectrophotometric measure (L*). Visual scores could not decipher this change. Also, in the age‐group 51–60 Y, forehead showed a decrease in redness. There was no change in skin yellowness. Visual evaluations and L* values in cheeks showed a steady skin darkening with age. Yellowness in the cheeks increased with age. This is in line with previous studies in Chinese, Japanese and Korean females showing increased darker tone and yellowness in cheeks with age.^2^, ^6^, ^8^, ^12^, ^21^, ^22^ A relatively low magnitude of change in the facial skin colour with age is in line with other studies involving Indians, African‐Americans, Caucasians and Mexican^9^ women but unlike a Chinese group from the USA^6^ and China,^7^, ^23^ where such effects were high. The skin evenness and radiance, in general, showed a steady decline with age. Hijab wearers showed a marginally lower visual scores, higher L* and a* and higher radiance and evenness, in majority of age‐groups. This is likely to have been contributed by photoprotection as hijab in face would obstruct some of the impinging radiation thus decreasing Sun induced tanning.

The aging parameters followed a faster course of change than that of colour. This is in line with response in eight cities from five Asian countries (China, India, Philippines, South Korea and Japan).^7^ Wrinkles and lines at crow's feet and the perioral area increased while skin elasticity decreased progressively with age. Krueger et al.^24^ showed an age‐related decline in the mechanical properties in German females of Fitzpatrick's skin type I‐IV. As indicated by Del Bino and Bernard^17^ for facial skin changes, the study population in this study being in the ITA range of 41 < ITA > 10 (intermediate, tan, and brown), showed higher pigmentary disorders and early onset of photoaging. Our study showed that in Indonesian females the pigmentary disorders are a major concern in line with other non‐Caucasian ethnicities.^6^

Of the five measured dermatological skin conditions, four showed an increase in severity with age; only PIH showed a lowered response with age. The prevalence of melasma in the Indonesian population in this study across age groups was high. In age groups 41–60, it was found to be 100%. Similar estimates in at corresponding age groups were ∼30% in Indian,^9^ Latino^25^ and oriental^26^ populations. Peak prevalence of seborrheic keratoses and solar/senile lentigines (∼65% and 37%, respectively) was like that of Indian population as studied across four cities (Mumbai, Delhi, Chennai and Kolkata).^9^ The estimates of these skin conditions were influenced by hijab status in age and condition‐specific manner. In general, majority of age groups wearing hijab was associated with a higher prevalence of solar and senile lentigines, seborrheic keratoses and PIH. Though the melasma prevalence did not change with respect to hijab status, the severity of melasma in hijab wearers was higher than non‐wearers in the age group of 31–40 Y (data not shown). Considering Sun exposure can have exacerbating effects on these skin conditions,^27^, ^28^, ^29^, ^30^ wearing hijab was anticipated to have mitigating effect as it can shield the face at least partly from the solar radiation. It may be that other life‐style factors influenced age hijab wearers for such higher propensity of skin disorders unless they are a chance occurrence.

Regarding prevalence of acne as a function of age, studies show the highest incidence in teenagers or young adults^30^ and a decreasing trend with increasing age.^31^, ^32^ A recent study has been conducted in the Indonesian population with the aim to delineate acne vulgaris clinical profile.^33^ In terms of prevalence, the highest was among female patients aged 20–24 Y (39%), followed by age group 15–19 Y (32.3%). The study also highlighted persistence of acne at age 35–39 Y (2.4%) and over 40 Y (3.3%). With these age‐acne prevalence data in the backdrop, the numbers derived from our study do follow an expected decline with each passing decade.

Skin elasticity and sebum levels showed a decline with age in line with a study of Chinese (Li et al.^8^) and Korean (Cho et al.^12^) women. A significant decline in TEWL indicated improvement in skin barrier function with age; the trend was like study with the Asian population in China, India, South Korea, Japan and the Philippines.^7^ At 160 μg.cm^−2^, sebum content in this study population was significantly higher than that reported in Chinese women^14^ and eight Asian cities^7^ (Harbin, Guangzhou and Shanghai in China, Calicut and New Delhi in India, Seoul in South Korea, Sendai in Japan, and Manila in the Philippines), although the trend of decline with age was similar. The sebum levels would also be determined by the time‐interval from preceding facewash. In a study where the gap between facewash and sebum measurement was similar (7 h), the sebum levels were also in similar zone.^34^

In conclusion, we captured the facial skin attributes and concerns in Indonesian females across age groups. The foremost among them are pigmentation and other skin conditions leading to uneven skin‐tone and attributes related to aging (skin elasticity, lines, and wrinkles) with progression of age. In general, the habit of wearing hijab provides some protection related to changes in skin colour while it was associated with a slightly higher prevalence of skin conditions. We believe the data on the prevalence and severity of the facial skin attributes and conditions across age groups presented in this study enriches knowledge of ethnic skin types and would be fundamental in designing mitigation approaches including the design of population‐specific cosmetic or therapeutic products in Indonesia.

## Data Availability

The data that support the findings of this study are available from the corresponding author upon reasonable request.
